# Cancer Detection Using an Artificial Secretable MicroRNA Found in Blood and Urine

**DOI:** 10.3390/biomedicines10030621

**Published:** 2022-03-07

**Authors:** Pei-Wei Shueng, Kuang-Chung Shih, Sanjiv Sam Gambhir, Deng-Yu Kuo, Hui-Yen Chuang

**Affiliations:** 1 Division of Radiation Oncology, Department of Radiology, Far Eastern Memorial Hospital, New Taipei 220, Taiwan; shuengsir@gmail.com; 2 School of Medicine, Faculty of Medicine, National Yang Ming Chiao Tung University, Taipei 112, Taiwan; 3 Division of Endocrinology and Metabolism, Department of Medicine, Cheng-Hsin General Hospital, Taipei 112, Taiwan; shihkc19610909@gmail.com; 4 Molecular Imaging Program at Stanford, School of Medicine, Stanford University, Stanford, CA 94305, USA; 5 Department of Radiology, Stanford University, Stanford, CA 94305, USA; 6 Department of Biomedical Imaging and Radiological Sciences, National Yang Ming Chiao Tung University, Taipei 112, Taiwan

**Keywords:** early cancer detection, liquid biopsy, tumor-activatable, miRNA

## Abstract

Biomarkers can potentially help in the detection and prognosis of diseases such as cancer, its recurrence, predicting response to therapy, and monitoring of response during and/or after treatment. Endogenous tumor blood biomarkers suffer from low concentrations that are not distinguishable from background noise and, if identified, the localization of the biomarker production site is not known. The use of exogenously introduced or artificial biomarkers can eliminate these issues. In this study, we show that cancer cells can be made to produce an artificial secreted microRNA (Sec-miR) that can be detected in media from cells in culture, and from both blood and urine in living mice. In culture, we show that chaining a number of Sec-miR sequences in a plasmid and transfecting cells with the plasmids could increase Sec-miR secretion as the number of sequences increases. Tumor induction in mice with a stably transfected HeLa cell line shows the presence and significant increase in the Sec-miR with time and tumor growth in plasma (*p* < 0.001, R2 = 0.5542). The relative half-life of the Sec-miR was seen to be 1.2 h in the plasma of living mice and was seen to appear in urine within 12 h. The transgene for the Sec-miR within a minicircle was introduced via the tail-vein into subcutaneous tumor-bearing mice. As the tumor growth increased with time, further in vivo transfection of the Sec-miR minicircles showed an increase in Sec-miR in both plasma and urine (R2 = 0.4546). This study demonstrated that an exogenous Sec-miR biomarker would allow for early tumor detection using in vitro diagnostics techniques.

## 1. Introduction

Cancer detection has relied on the production of endogenous tumor-specific biomarkers that can be detected in body fluids, mostly in the blood. This has proven to be difficult due to the variability of the normal concentration of biomarkers within a patient population and a background level of biomarkers due to normal cells and confounding disease states that prevent the accurate differentiating of an increased biomarker level due to the presence of a tumor [1,2]. This has prevented the introduction of new biomarkers into patient care, even though a relatively high number of them are being discovered [3]. To overcome the above stated limitations of endogenously present biomarkers, we have previously demonstrated the use of an exogenously introduced gene encoding for a protein biomarker that allows for the detection of tumors in living mice. The protein was introduced as a transgene within a minicircle. Minicircles are plasmids that are void of the bacterial backbone, leading to a smaller size with less immunogenicity and higher rate of transfection [4,5]. They have been studied mostly in the field of gene therapy. Minicircles have been shown to be transcribed episomally without integration into the genome, giving the added safety benefits of non-integration combined with the non-immunogenicity for eventual clinical translation [6,7].

We have generated minicircles that are driven by a tumor-activatable Survivin promoter (pSURV), so that only tumor cells can express the downstream protein that is secreted and detected in the blood. Moreover, we have demonstrated their use in detecting cancers by harboring a sequence for a secretable enzyme as a reporter, which can be non-invasively detected in blood [8]. Although drawing blood is a minimally invasive and relatively easy procedure, repeated blood sampling could be uncomfortable and challenging to some patients. Detecting biomarkers present in the urine may solve these problems. However, there are fewer protein biomarkers found in the urine than in the blood because of the molecular weight cutoff (around 30–50 kDa) for glomerular filtration [9]. Currently, protein-based urinary biomarkers are mostly used for identifying cancer which originated from the urogenital tract [10,11,12]. In contrast, nucleic acid-based biomarkers including cell-free DNA or RNA (mRNA and microRNA) detected in urine can be used for distinguishing types of cancer originating outside the urogenital tract from lung cancer [13], breast cancer [14,15,16], and colorectal cancer [17]. These results demonstrate that circulating cell-free nucleic acids have potential as both blood and urinary biomarkers.

MicroRNAs are small (22-nucleotides) non-coding RNAs that are found in a relatively stable form in bodily fluids when complexed with proteins or in exosomes [18]. Their stability allows them to have a longer half-life and be easily detected once released into circulation. We have also previously studied an artificial secreted microRNA (Sec-miR) as a secreted reporter from cancer cells in culture and in living subjects with the use of cells stably transfected with the gene for Sec-miR [19]. The sequence of Sec-miR is not found naturally and its nucleotide structure makes it non-immunogenic. The detection of Sec-miR by quantitative reverse transcriptase polymerase chain reaction (qPCR) could amplify its signal further and lead to increased analytical sensitivity and earlier cancer detection when used as a reporter, compared to proteins.

In the current study, we modified the Sec-miR-encoding tumor-activatable minicircles with chaining strategy to increase detection sensitivity and to demonstrate that Sec-miR could function as both blood and urine biomarkers for distinguishing tumor-bearing from healthy mice. We also demonstrate that the linking of multiple copies of Sec-miR within the plasmid allows for higher detection sensitivity of the miRNA secreted from transfected cells. The detection of Sec-miR from the urine allows for an easy non-invasive early tumor detection method which, together with the Sec-miR plasma levels, can determine the relative size of the tumor. Our diagnostic method suggests the use of simple non-invasive methodology of early cancer detection using exogenous reporter biomarkers.

## 2. Materials and Methods

### 2.1. Plasmid and Minicircle Construction

All sec-miRNA constructs were built using standard PCR and cloning methods and sequenced by Sequetech (Mountain View, CA, USA). Both tumor-specific sec-miRNAs, parental plasmids (PP) and minicircle (MC), were generated using the system (System Biosciences, Mountain View, CA, USA) proposed by Kay et al. [20]. Secreted embryonic alkaline phosphatase (SEAP) in the PP-pSurv-SEAP-WPRE plasmid was removed by restriction enzyme digestion, and 12 copies of Sec-miRNA were inserted using In-fusion cloning technology (Clontech, Mountain View, CA, USA) to generate PP-pSurv-Sec-miR 12X-WPRE. Both PP-pSurv-Sec-miR 12X-WPRE (PP) and MC-pSurv-Sec-miR 12X-WPRE (MC) were produced according to the protocols proposed by Kay et al. Briefly, ZYCY10P3S2T E. coli were transformed with PP, colonies were picked, inoculated into kanamycin-containing TB broth and grown overnight. MC induction was initiated by adding an equal volume of antibiotic-free LB broth with 0.1% L-arabinose and 4% 1N NaOH, and grown for another 5.5 to 6 h at 30 °C. Both PP and MC were isolated using endotoxin-free maxipreps (Zymo Research, Orange, CA, USA).

### 2.2. Cell Culture, Transfection and Stable Clone Establishment

Hela cells were maintained in DMEM (Gibco, Carlsbad, CA, USA) supplemented with 10% fetal bovine serum and antibiotic–antimycotic solution and incubated in humidified 5% CO_2_ incubator at 37 °C.

Then, 5 × 10^4^ Hela cells per well were seeded in a 24-well plate and transfected with different constructs using the same mass (1 µg) of DNA and an equal amount (2 µL) of Lipofectamine 2000 (Invitrogen, Carlsbad, CA, USA) following the manufacturer’s protocols. Medium was changed 1 day after transfection and kept for another 2 days. Media were collected, centrifuged at 1000 rpm for 3 min to remove cell debris, and the supernatants were utilized for Sec-miR analysis using qPCR.

Next, 5 × 10^4^ Hela cells per well were seeded in a 24-well plate and transfected with 1 µg of pEF1-Luc2-Sec-miR 8X and 2 µL of Lipofectamine 2000 as described. Transfected cells were selected with medium containing 2 µg/mL blasticidin and the expressions of luciferase and Sec-miR were determined by bioluminescent imaging and qPCR, respectively.

### 2.3. Sec-miR Injection and Clearance Studies

**Figure A1:**
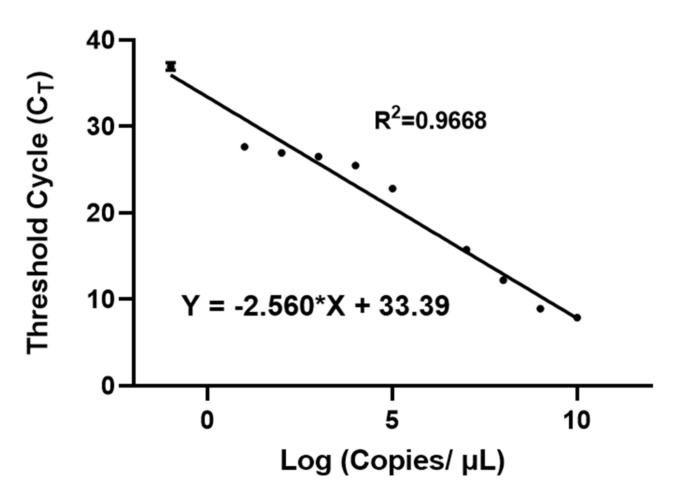
qPCR standard curve of Sec-miR. Different copy numbers of synthesized Sec-miR were spiked in 200 µL culture medium. The Sec-miR-containing medium was further processed with an RNA isolation kit and subjected to qPCR assay to generate a qPCR standard curve of Sec-miR. The X-axis is shown as the log 10 of the copy number per µL.

At 80–90% confluency, the FBS-containing media were removed, replaced with serum-free media, and the Hela/pEF1-Luc2-Sec-miR 8x stable cells were left to grow in serum-free medium for two days. Conditioned media from two flasks were collected, centrifuged, and concentrated. Then, 200 µL of concentrated Sec-miR containing media was injected into each mouse. The concentration of concentrated Sec-miR was calculated using the standard curve shown in Figure A1, and was about 10^8^ copies/µL. The first blood sample was collected from the submandibular vein within 2 min after injection and the blood sample was collected every 12 h for 2 days. All the mice were kept in the metabolic cages from 12 h before injection to 2 days after injection, and all urine samples from every 12 h were pooled and centrifuged. Both blood and urine samples were stored at −80 °C.

### 2.4. Mice Tumor Models and Tumor-Specific Sec-miR MC Injection

All procedures performed on animals were approved by Stanford University’s Institutional Animal Care and Use Committee and were within the guidelines of humane care of laboratory animals (Protocol No. 26448). All animal experiments were repeated twice. Then, 1 × 106 Hela or Hela/pEF1-Luc2-Sec-miR 8X cells were implanted into the right thighs of 6- to 8-week-old female nude mice (Nu/Nu; Charles River, Wilmington, MA, USA) to generate a subcutaneous tumor model. Mice were kept in metabolic cages (one mouse per cage) five nights per week after tumor injection. Tumor sizes were measured with calipers (mm^3^; width2 × length × 4/3π), and BLI imaging was performed after intraperitoneal injection of D-Luciferin (30 mg/mL; 4.5 mg total dose) using an IVIS 200 imaging system (PerkinElmer, Waltham, MA, USA).

Another group of Hela tumor-bearing mice were tail-vein-administered 40 µg of tumor-specific Sec-miR MC complexed with in vivo jetPEI (N/P ratio = 8, Polyplus, Illkirch, France) and re-suspended in 200 µL of 5% glucose when average tumor sizes reached 150 mm^3^. Additionally, healthy normal mice also received MC injection, and tumor-bearing groups of irradiated mice were administered 200 µL of 5% glucose alone, serving as control groups.

### 2.5. Sample Collections and Preparations

Blood samples were collected from the submandibular vein once a week and 2 days after each MC injection for the mice with subcutaneous Hela tumor. Next, 100–200 µL blood was collected in K3-EDTA-coated tubes (Greiner, Kremsmünster, Austria), kept on ice, and centrifuged at 1000 g for 10 min at 4 °C. After tumor implantation, mice with subcutaneous tumors were kept in metabolic cages 8 h per day for 5 weeks. Urine samples from each mouse were collected and spun down at 1000 g for 10 min. Samples collected within the same week were pooled and extracted for analysis. Both blood and urine samples were stored at −80 °C prior to RNA isolation.

### 2.6. RNA Isolation and qPCR

Total RNA including miRNA was isolated using the miRNeasy Serum/Plasma kit (Qiagen, Hilden, Germany) from cell medium, plasma, and urine. Urine samples from each mouse were pooled every week and concentrated with a 100 kDa Amicon filter tube (Millipore, Burlington, MA, USA) down to ~200 µL. RNA was extracted from 200 µL medium or concentrated urine samples and 50 µL plasma samples per prep and eluted with 14 µL nuclease-free water. Next, 5 µL of total RNA was used for RT-PCR with the TaqMan Small RNA assays (Applied Biosystems, Foster City, CA, USA) in 15 µL total reaction mixture. The miRNeasy Serum/Plasma Spike-In Control (cel-miR-39) was used to normalize RNA isolation efficiency. Sec-miR expressions were assessed with the TaqMan MicroRNA assay (Applied Biosystems, Foster City, CA, USA) with the custom primer specific for the Sec-miR sequence (AAAUGUACUGCGCGUGGAGAC). Each 20 µL total PCR mixture contained 1.33 µL of the RT product, 1 µL of 20X primer, and 10 µL of TaqMan 2X Universal PCR Master Mix. qRT-PCR was performed on the iCycler Real-Time PCR detection system (Bio-Rad, Hercules, CA USA).

### 2.7. Statistical Analysis

All statistical analysis was performed with GraphPad Prism 9.3.0 software. All the data were presented as mean ± SD. Comparisons of data between two groups was performed using Student’s *t*-test. Comparisons of data between more than two groups were performed using one-way ANOVA followed by Tukey’s post hoc multiple comparisons test. Correlations among tumor sizes and Sec-miR expressions in plasma and urine samples were also analyzed. For all tests, a *p*-value less than 0.05 was considered to be statistically significant.

## 3. Results

### 3.1. Sec-miR Chaining Strategy Significantly Increases Sec-miR Expressions In Vitro

The aim of the study was to create a tumor-specific biomarker, which can be detected in blood and urine, for detecting cancer non-invasively. In our previous study [19], we have shown that Sec-miR expression from cells can be amplified by increasing the copy numbers of Sec-miR in a single construct. Thus, we made various constructs with multiple copies of Sec-miR driven by the same promoter to increase the Sec-miR levels from cells. Vector sizes can influence the transfection efficiency. The size of Sec-miR pre-miRNA is 132 nucleotides, allowing us to increase the copy numbers up to 12 and build a reporter whose size is comparable to SEAP used in our previous study [8].

**Figure 1:**
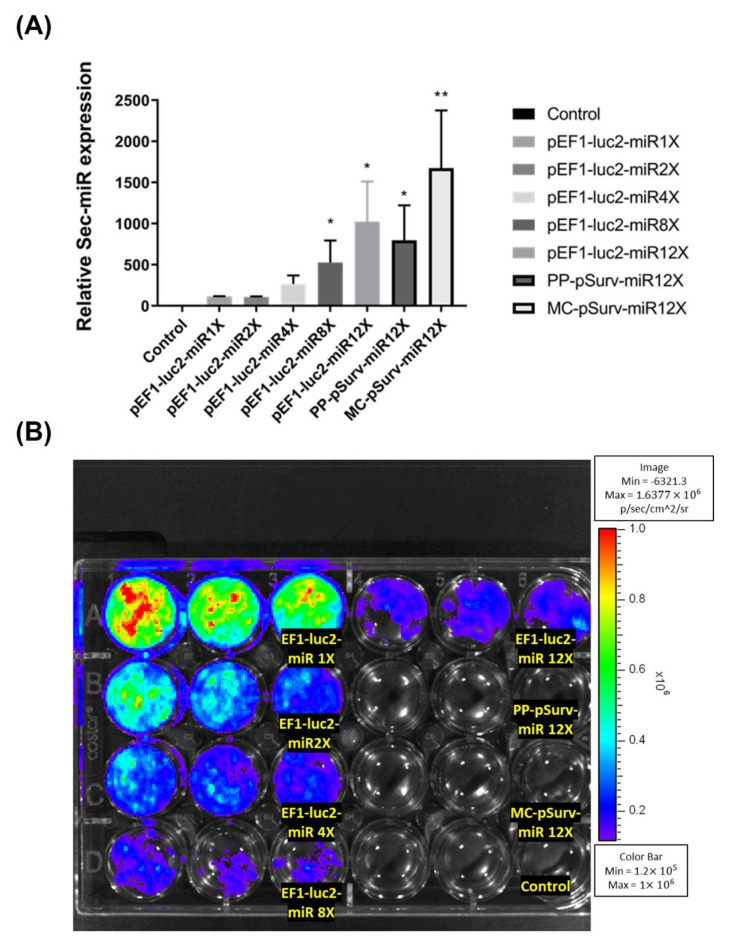
Sec-miR reporter expression from cells could be amplified by inserting multiple copies of Sec-miR into a single construct. Hela cells were transiently transfected with plasmids containing 1, 2, 4, 8, or 12 copies of Sec-miR driven by constitutive EF1 promoter or tumor-specific Survivin promoter (pSurv) in different wells. (**A**) All the Sec-miR expressions were normalized to the values in wells transfected with the 1X Sec-miR copy plasmid. Data are expressed as mean ± SD. Sec-miR expressions were detected in all the wells transfected with Sec-miR constructs; the Sec-miR expressions significantly increased as the copy numbers of Sec-miR increased in the plasmids. Moreover, Sec-miR expressions in the cells transfected with PP-pSurv-Sec-miR 12X-WPRE and MC-pSurv-Sec-miR 12X-WPRE were also compared. Sec-miR expressions of MC-transfected cells were significantly higher than PP-transfected cells and were comparable to the expressions detected in the cells transfected with pEF1-Luc2-Sec-miR 12X. (**B**) Bioluminescent signals emitted from cells transfected with different constructs were acquired after medium collection. *, *p* < 0.05; **, *p* < 0.01.

Increasing copy numbers of Sec-miR in a single construct from 1 to 12 significantly (*p* < 0.05) enhanced Sec-miR expression in the media of transient transfected Hela cells (Figure 1A). Interestingly, amplifications of Sec-miR expressions seem to be linear instead of power of 2 increases. Moreover, the Sec-miR expressions of MC-pSurv-Sec-miR 12X-WPRE are similar to the level of pEF1-Luc2-Sec-miR 12X and significantly higher (*p* < 0.05) than PP-pSurv-Sec-miR 12X-WPRE. The bioluminescent signals emitted from transfected cells were also acquired after medium collection (Figure 1B).

### 3.2. Sec-miR Serves as Liquid Biomarker for Cell Monitoring In Vivo

**Figure 2:**
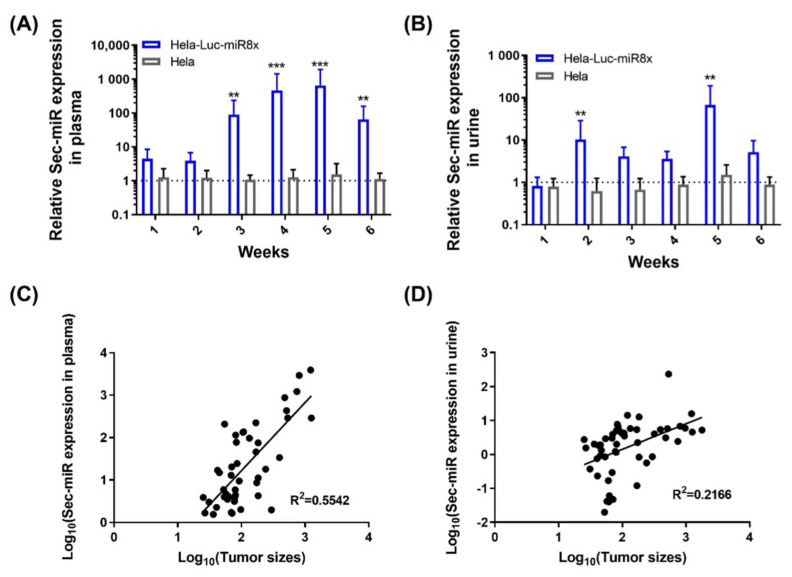
Sec-miR is detectable in the blood and urine of mice bearing Sec-miR-expressing Hela tumors and can be used as a complement to imaging reporters. Both parental Hela and the Hela cells stably express Luc2 and 8 copies of Sec-miR (Sec-miR 8X) were inoculated to mice; blood and urine of these tumor-bearing mice were collected and analyzed. Blood samples were collected once a week after the average tumor size reached 150 mm^3^. (**A**) Significantly higher Sec-miR expressions were detected in blood from mice with Hela/EF1-Luc2-Sec-miR 8X tumors, and the Sec-miR expression increased as the tumors grew. (**B**) Detectable Sec-miR expressions were found in urine from the mice with Hela/EF1-Luc2-Sec-miR 8X tumors from week 2 after tumor inoculation. The Sec-miR expressions did not increase as the tumor grew but varied over time. Correlations of tumor sizes and Sec-miR expressions in (**C**) blood and (**D**) urine. Data from Hela/EF1-Luc2-Sec-miR 8x mice are normalized to data from parental HeLa mice and expressed as mean ± SD. **, *p* < 0.01; ***, *p* < 0.001.

**Figure A2:**
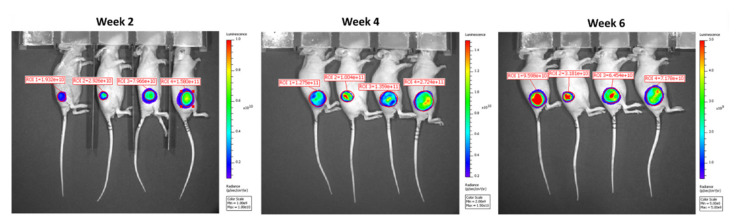
Representative BLI images of Hela/pEF1-Luc2-Sec-miR 8X mice. BLI images were acquired on weeks 2, 4, and 6 after tumor inoculation. Viable cells present in tumors seemed to decrease as the BLI signal reduced on Week 6 after tumor inoculation.

Hela and Hela/pEF1-Luc2-Sec-miR 8X cells were implanted subcutaneously into the right thighs of female nude mice. Mice were kept in metabolic cages around 8 h a day after tumor implantation. Tumor growths were tracked by caliper measurement. Plasma samples were collected on the same days after tumor measurement. Surprisingly, Sec-miR expressions were detectable in the blood samples collected from Hela/pEF1-Luc2-Sec-miR 8X mice at the first time point (Day 7 after tumor implantation). The averaged Sec-miR signals were 4- to 5-fold higher than the signals from Hela tumor mice (Figure 2A). The signals elevated as tumor sizes increased from week 2 to week 4 and declined when tumors likely became necrotic in week 6 (Figure A2). Detectable Sec-miR expressions in urine samples from Hela/pEF1-Luc2-Sec-miR 8X mice were first found in the samples collected in week 1 after tumor inoculation. Instead of increasing as tumors grew, the Sec-miR expressions were relatively stable throughout the whole experiment (Figure 2B). The correlations of tumor size and Sec-miR expression in blood (Figure 2C) and urine (Figure 2D) were established. Sec-miR expressions in blood seemed to be an ideal reporter for tumor growth monitoring. A positive correlation was found between tumor sizes and Sec-miR expressions in blood (R2 = 0.5542, Figure 2C); however, there was no strong correlation between tumor sizes and Sec-miR expressions in urine (R2 = 0.2166; Figure 2D). The results imply that Sec-miR detected in blood and urine may be used as biomarkers to determine the presence of tumor, although Sec-miR levels may not reflect the size of tumor burden.

### 3.3. Sec-miR Has a Short Half Life In Vivo

**Figure 3:**
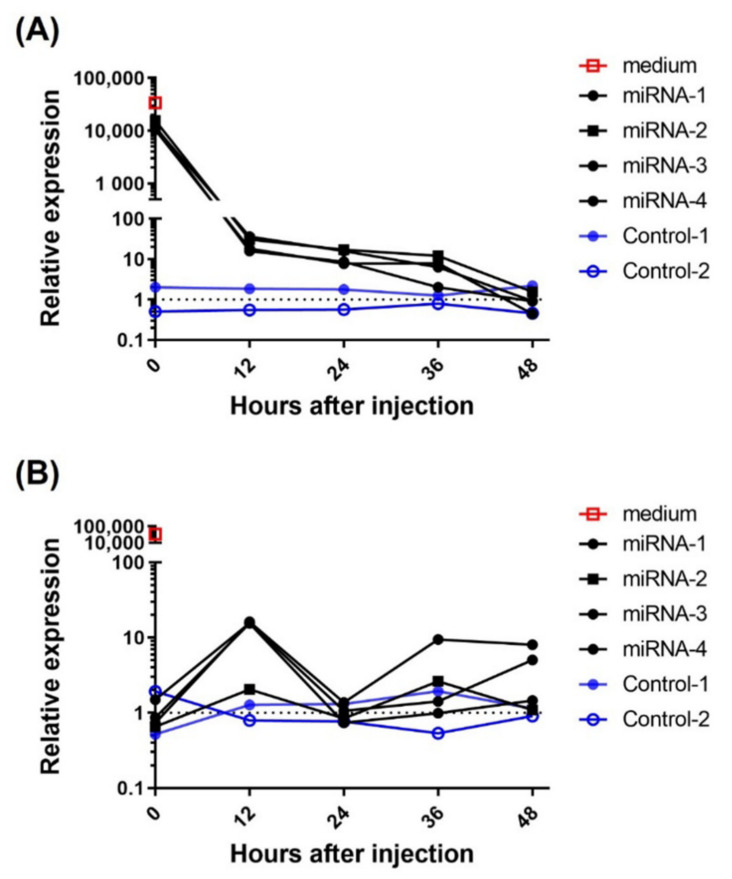
miRNA clearance in vivo. Normal mice were intravenously injected with 200 µL concentrated Sec-miR containing medium. The blood and urine samples were collected every 12 h for two days to determine the Sec-miR clearance in vivo. (**A**) Around 25% of Sec-miR expression was detected in the first blood sample (within 2 min after injection) compared to the injected Sec-miR-containing medium. Sec-miR expression in blood was significantly dropped in the first 12 h after injection and remained detectable until 36 h after injection. (**B**) Three of four Sec-miR injected mice showed around 10-fold expressions of Sec-miR in the first 12 h (0–12 h) urine samples. Decreased Sec-miR expressions were found in the second (12–24 h) and fourth (36–48 h) 12 h time period and came back in the third 12 h after injection.

The Sec-miR expressions in blood showed a different pattern as compared to that in urine as previously described. The clearance of Sec-miR experiment was performed to understand the half-life and metabolism of Sec-miR in vivo. Microvesicle encapsulations and argonaute proteins make miRNAs more stable than other RNA in vivo. Thus, conditioned media from Hela/pEF1-Luc2-Sec-miR 8X cells were collected, concentrated, and systemically injected into mice to mimic in vivo conditions. As shown in Figure 3A, around 25% of the injected dose could be detected in blood at the first time point (within 2 min after injection), and the Sec-miR signals in blood were tracked for 48 h by drawing blood every 12 h. A significant drop in Sec-miR signal in blood was observed in the first 12 h, and the Sec-miR signals detected at the 12 h time point were about 0.1% of the signals detected at the first time point. The Sec-miR signal in blood remained at a similar level (10-fold higher than the signals detected in the normal mice) until 36 h after injection. The Sec-miR signals in blood decreased to the background 48 h after injection. Figure 3B shows the Sec-miR signal changes in urine. Unlike blood samples that were collected once every 12 h, the urine samples from every 12 h were pooled, concentrated, and analyzed. The Sec-miR signals in urine were first detected in the first 12 h period, about 10-fold higher than the signals from the normal mice. Interestingly, the signals decreased in the second 12 h, and increased again in the third 12 h after injection.

### 3.4. Tumor-Bearing Subjects Could Be Differentiated from Healthy Controls Using Systemic Administration of Tumor-Specific Sec-miR Minicircles

Our ultimate goal in this study was to verify whether systemic tail-vein administration of tumor-specific Sec-miR MC could be used for detecting cancer non-invasively. Hela human cervical cancer cells were administered subcutaneously to generate a tumor-bearing model (*n* = 5). Tumor-specific Sec-miR MC were given systemically when the average tumor size reached 150 mm^3^.

**Figure 4:**
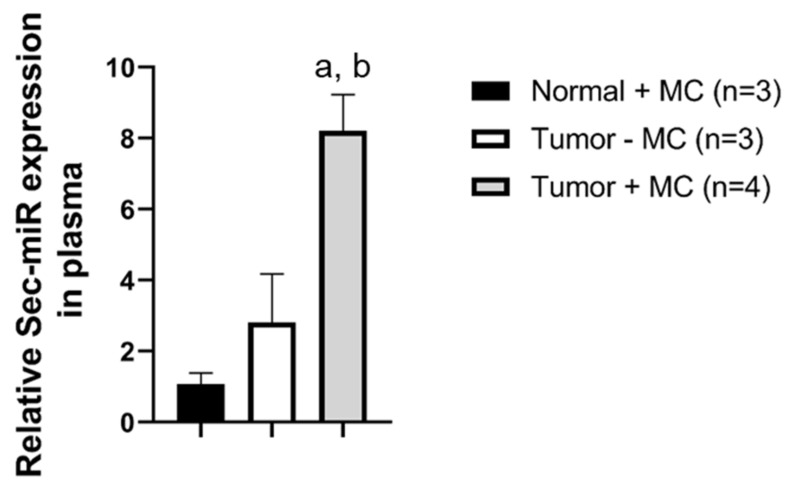
Systemic delivery of tumor-specific Sec-miR MC allows the identification of tumor-bearing subjects. After the average tumor size reached 150 mm^3^, tumor-specific Sec-miR MCs (40 µg, N/P = 8) were injected systemically to tumor-bearing mice (Tumor + MC) and healthy normal mice (Normal + MC). Normal (tumor-free) mice received MCs (Normal + MC), and tumor-bearing mice that did not receive MCs (Tumor − MC) were served as control groups. Sec-miR expressions in blood were measured on day 2 after MC injection. Sec-miR expressions significantly increased in blood compared to Normal + MC and Tumor − MC groups. a, compared to Normal + MC, *p* < 0.01; b, compared to Tumor-MC, *p* < 0.05.

Sec-miR levels in the blood and urine samples were measured on day 2 and days 1, 2, 3, 5 after MC injection, respectively. Our previous study [8] showed that the Survivin promoter has some leakiness and could result in slightly positive signals from normal tissues. Thus, normal mice also received MC as one of the control groups to show the background signals caused by MC injection (Normal + MC; *n* = 3). Another control group was tumor-bearing mice without MC injection (Tumor − MC; *n* = 3). The Sec-miR expression could only be detected in plasma (Figure 4) and the signals obtained from the tumor-bearing mice with MC injection (Tumor + MC, *n* = 4) were significantly higher than that of the Normal-MC and Tumor-MC groups. However, the signals could not be detected in urine samples, even though the urine samples were collected for over 12 h.

## 4. Discussion

Cancer mortality rates are strongly related to the stage at diagnosis. Additionally, it is well-known that some cancer types, including breast cancer, colorectal cancer, and ovarian cancer, are usually curable if they are caught at the early stage with the right medical intervention. Strikingly, cancer mortality rates are significantly higher in less-developed countries than developed countries, which may result from the lack of advanced medical instruments and well-trained personnel. Furthermore, the late diagnosis caused by unavailable inexpensive and simple cancer screening methods also leads to increased cancer mortality. Our group previously demonstrated that tumors could exist in the body for many years before being detected by current laboratory and imaging examinations [3]. Therefore, better cancer detection strategies are urgently needed.

Liquid biopsy detecting endogenous biomarkers such as protein-based biomarkers (e.g., PSA and CA-125), ctDNA and circulating tumor cells (CTCs) that shed from the primary tumors has showed promise in cancer detection. However, the low frequencies or high background signals of these endogenous biomarkers limit the detection sensitivity of liquid biopsy. Instead of detecting endogenous biomarkers mentioned above, we previously demonstrated that exogenously introduced genetically encoded biomarkers could identify tumor-bearing mice from healthy subjects by measuring the levels of exogenous artificial biomarkers in the blood [8]. Being regulated by the tumor-specific Survivin promoter (pSURV), the expression of the exogenous artificial biomarker eliminates the current limitations of variability of background levels as well as tumor heterogeneity. Even though the systemic leakiness of the Survivin tumor-specific promoter has been mentioned [21,22], the overall background levels from normal tissues were relatively negligible. Other tumor-activatable promoters could also be studied that have less leakiness such as XRCC2 [23] or hTERT [24,25] in the future.

The current study shows that Sec-miR could be an ideal blood and urinary biomarker for cancer detection. A positive correlation between tumor sizes and plasma Sec-miR level was found in mice injected with Hela cells stably expressing Sec-miR (Figure 2C), and therefore would potentially allow for the assessment and study of disease progression and therapy response. Even though there is no correlation between tumor size and the amount of Sec-miR found in urine, it is sufficient as a method of determining the presence of a tumor.

The use of the unique miRNA sequence has several functional advantages as an exogenous artificial reporter, e.g., being not found in blood or tissues, non-immunogenic, highly stable in blood and having no biological function. The inherent amplification of the method of detection leads to higher sensitivity than protein biomarkers. Future studies will also look into more sensitive detection methods such as droplet digital PCR [26], graphene-based biosensors [27], and chemiluminescent detection strategies [28,29]. These strategies have been shown to push the detection limit to nanomolar or even attomolar levels [30], which may further enhance the diagnostic power of miRNA-based liquid biopsy. Furthermore, multiplexed detection of miRNAs can be achieved by using CRISPR/Cas9 [31,32] or CRISPR/Cas12a [33] techniques, surface-enhanced Raman scattering (SERS) biosensors [34], and the biotin-neutravidin reaction [35]. Multiplexed miRNA detection may help further distinguish the origin of cancer and elevate detection specificity. For instance, identification of miR-33a-5p, miR-128-3p [36], and Sec-miR levels could be applied for lung cancer detection. Prostate cancer may be found when simultaneously detecting has-miR12-5p, has-miR-198 [37] and Sec-miR.

The clearance of injected Sec-miR into mice from blood was seen to be rapid and decreased about 1000-fold within the first 12 h (Figure 3A). This may prove to be a disadvantage to the use of this Sec-miR as a blood biomarker when compared to other proteins with longer half-lives such as SEAP. According to the literature, mature miRNAs are relatively stable molecules with a half-life from hours to days. Both the biogenesis and decay of miRNAs are highly regulated, and the miRNA degradation is controlled by multiple factors, including the status of cells, cell cycle, and degrading enzymes (exoribonucleases) [38]. Since binding to Argonaute and exosome encapsulation could increase the stability of miRNA, future studies would also need to determine if the Sec-miR is sequestered within an exosome or attached to a nucleoprotein. Additions of methyl groups and a poly-A tail to the 3′ ends of miRNA have been found to stabilize miRNAs [39]; other sequences of artificially secreted miRNAs would need to be investigated to determine if the stability could be increased. Zhou and colleagues also demonstrated that the stability and temporal expression pattern are strongly affected by the first nucleotide of the miRNA [40]. Hence, simultaneous expressions of Sec-miR and argonaute proteins or modification of 5′ nucleotide on the Sec-miR sequence may help extend the half-life of Sec-miR in living subjects.

As shown in Figure 2B and Figure 3B, we observed drops and rises in Sec-miR in urine samples. The fluctuations of the urinary Sec-miR signal may result from the way that we collected urine samples. Mice were put into the metabolic cages separately (i.e., one mouse per cage) and injected with 200 µL sterile PBS intraperitoneally before the urine collection. However, the urine collection was conducted in the daytime when mice were less active. Accordingly, the amounts of urine and the molecules contained in it would be different and potentially lead to the fluctuation of urinary Sec-miR signals. The clearance of the plasma Sec-miR into the urine seems to be not correlated with tumor size or plasma concentration. Several studies have observed that some miRNA levels are reduced in the circulation but increased in the urine of patients with impaired renal function [41,42]. These findings imply that the glomerular filtration rate and renal function might affect the excretion of miRNAs, but it may also relate to pathogenesis progression. At the same time, there could be a certain regulatory mechanism that limits the amount of Sec-miR in the urine and this would need to be studied further.

The tumor-activatable minicircles system encoding the Sec-miR could successfully distinguish tumor-bearing mice from non-tumor-bearing mice by a non-invasive analysis of plasma (Figure 4). These results indicate the possibility of using these Sec-miR-encoding tumor-activatable minicircles to identify whether a subject has tumors or not through blood-based liquid biopsy. Ideally, the tumor-activatable minicircles will be systemically given to patients after being complexed with transfection agents. The liquid biopsy will be performed before and after minicircle injection, and the characteristics of utilized biomarkers will determine the sample collection time points. The changes in biomarker level (after–before) will be calculated to recognize cancer patients. The expressions of unique Sec-miR sequences or other artificial biomarkers are regulated by the tumor-specific Survivin promoter, ensuring that artificial biomarkers will only be shed from tumors. These exogenously expressed biomarkers make tumor detection highly specific. Expressions of endogenous tumor biomarkers often are not limited to cancer cells but other non-cancerous healthy conditions, such as elevated PSA, found in prostatitis. Increased tumor sizes or radiopharmaceutical uptakes detected by medical imaging may not be caused by tumor growth, but immune cell infiltration-related pseudo-progression. Therefore, the tumor-activatable minicircle-encoded artificial biomarkers may provide higher tumor-specificity with minimal background signals compared to the endogenous tumor biomarkers and medical imaging examination. Liquid biopsy generally cannot provide any anatomical information and it is hard to identify the origin of these detected cancer cells. The combination of the Sec-miR together with imaging reporters for PET or MRI would allow for localization of the tumors for determining subsequent treatments and monitoring tumor growth.

Compared to other gene delivery strategies, minicircles are episomal non-viral vectors with minimal gene integration potential. Removal of prokaryotic sequences reduces potential immunogenicity and gene silencing, which is sometimes observed in cells transfected with traditional parental plasmids. With smaller vector sizes, transfection with minicircles may have higher transfection efficiency than the parental plasmids encoding the same transgenes [4]. The efficiency of the system is also dependent on the transfection agent used. There are a variety of agents that might be superior to currently used in vivo jetPEI in transfection that may increase the introduction of the minicircles into the cells [43,44,45] and increase the levels of biomarker expressed by the transfected cells. Nanoparticles with tumor-targeting capabilities are widely studied and a combination of tumor-targeting nanoparticles and tumor-activatable Sec-miR-encoding MCs may lead to higher tumor specificity. Future studies will focus on increasing targeted specificity. This will also decrease the background resulting from promoter leakiness when the minicircles are not targeted only to the tumor cells. The success of transfection is highly dependent on the uptakes of complexes formed by DNA and transfection agents. Therefore, the current strategy would be more suitable for detecting solid tumors than hematological malignancy because of the enhanced permeability and retention effect (EPR effect) [46] and limited lymph circulation present in the solid tumors. Additionally, artificial biomarkers generated by hematological malignancies might be diluted faster and become undetectable in circulation in a short time than solid tumors.

This study determines the use of an exogenous artificial Sec-miR as a biomarker for tumor detection by introduction into the cells via a non-viral episomal minicircle. The Sec-miR signals could be detected in both plasma and urine in mice bearing Sec-miR-expressing Hela tumors, allowing for an easy non-invasive detection. Furthermore, the Sec-miR signals could be significantly enhanced by the chaining strategy and enhance detection sensitivity. Most importantly, the current study shows the potential of using pSURV-driven Sec-miR encoding minicircles for cancer detection with blood samples. These tumor-activatable minicircles encoding exogenous biomarkers could provide a sensitive method for tumor detection as well as tumor recurrence or tumor treatment.

## Data Availability

The data presented in this study are available on request from the corresponding author. The data are not publicly available due to currently applying for a patent.
